# Cell surface marker heterogeneity in human myeloma cell lines for modeling of disease and therapy

**DOI:** 10.1038/s41598-024-80263-y

**Published:** 2024-11-20

**Authors:** Alenka Djarmila Behsen, Toril Holien, Francesca Micci, Morten Rye, Jenny Malm Rasmussen, Kristin Andersen, Eli Svorkdal Hess, Magne Børset, Jonathan Keats, Thea Kristin Våtsveen, Kristine Misund

**Affiliations:** 1 Department of Clinical and Molecular Medicine, Norwegian University of Science and Technology (NTNU), Trondheim, Norway; 2 Department of Biomedical Laboratory Science, Norwegian University of Science and Technology (NTNU), Trondheim, Norway; 3 Department of Immunology and Transfusion Medicine, St. Olav’s University Hospital, Trondheim, Norway; 4 Department of Hematology, St. Olav’s University Hospital, Trondheim, Norway; 5 Section for Cancer Cytogenetics, Institute for Cancer Genetics and Informatics, The Norwegian Radium Hospital, Oslo University Hospital, Oslo, Norway; 6 Clinic of Surgery, St. Olav’s University Hospital, Trondheim, Norway; 7 BioCore - Bioinformatics Core Facility, Norwegian University of Science and Technology (NTNU), Trondheim, Norway; 8 Clinic of Laboratory Medicine, St. Olav’s University Hospital, Trondheim, Norway; 9 Integrated Cancer Genomics Division, Translational Genomics Research Institute (TGen), Phoenix, AZ USA; 10 K.G. Jebsen Centre for B Cell Malignancies, Institute of Clinical Medicine, University of Oslo, Oslo, Norway; 11 Institute of Clinical Medicine, Department of Immunology, Oslo University Hospital, Oslo, Norway; 12 Precision Immunotherapy Alliance, University of Oslo, Oslo, Norway; 13 Department of Medical Genetics, St. Olav’s University Hospital, Trondheim, Norway

**Keywords:** Myeloma, Experimental models of disease

## Abstract

Multiple myeloma (MM) is a hematological malignancy originating from plasma cells. Genetically, MM is categorized into two subtypes: hyperdiploid and non-hyperdiploid tumors, with distinct chromosomal characteristics. Human myeloma cell lines (HMCLs) are instrumental in understanding MM and identifying therapeutic targets. However, their utility depends on their resemblance to patient-derived cells. We analyzed protein expression of cell surface markers, including myeloma drug targets and immunotherapy-relevant markers, in nine HMCLs using flow cytometry. RNAseq analysis was performed to correlate protein expression with transcriptomic data. While five cell surface markers (CD47, CD49d, CD138, CD269 (BCMA), and GPRC5D) were highly expressed and three (CD19, CD20, and CD117) were low or negative across cell lines, heterogenous expression was observed for the 16 remaining markers. A comparative study with patient-derived transcriptomic data indicated that four of our six in-house HMCLs closely resembled patient disease. Furthermore, our hyperdiploid HMCLs correlated better with hyperdiploid than non-hyperdiploid patient samples. Here, we also describe three previously uncharacterized HMCLs; IH-1, URVIN and FOLE. Our findings underscore the importance of evaluating surface protein expression in HMCLs when modeling MM. The observed variations in expression levels emphasize the need for a strategic selection of cell lines based on the study’s objectives.

**Supplementary Information:**

The online version contains supplementary material available at 10.1038/s41598-024-80263-y.

## Introduction

Multiple myeloma (MM) is the second most common hematological malignancy, originating from plasma cells in the bone marrow^1^. Genetically, MM is divided into two distinct subtypes. Approximately half of MM patients have hyperdiploid tumors, with > 47 or < 75 chromosomes in the cancer cells. These tumors are characterized by multiple trisomies of chromosomes 3, 5, 7, 9, 11, 15, 19, and 21, and lack of recurrent immunoglobulin gene translocations. The other half have non-hyperdiploid tumors, with < 46 or > 75 chromosomes, and frequent chromosome translocations involving the immunoglobulin enhancers, the most common being t(4;14), t(14;16), t(14;20), t(6;14) and t(11;14)^2^.

Cancer cell lines are widely used as disease models in cancer research. Human myeloma cell lines (HMCLs) are used extensively to gain insight into MM disease mechanisms, and to identify new therapeutic targets. The usefulness of HMCLs is largely dependent on how well they reflect patient myeloma cells. Given the heterogeneity of MM tumors, relying on results from a single HMCL is inadequate. Furthermore, the choice of cell lines for a given study must be strategic, based on the gene or protein of interest. Although approximately half of MM patients have hyperdiploid tumors, most cell lines are derived from non-hyperdiploid patient clones. Since very few hyperdiploid cell lines exist, studies that have evaluated their usefulness to model patient disease are lacking.

**Fig. 1:**
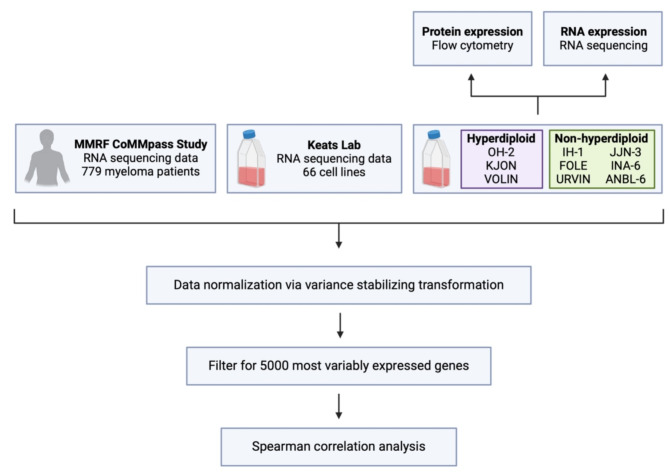
Overview of experimental plan. Flow cytometry and bulk RNA sequencing was used to analyze protein surface and RNA expression levels of relevant drug-related targets, including molecules relevant for immunotherapy. RNA sequencing data from these cell lines was compared with sequencing data from 779 newly-diagnosed myeloma patients from the CoMMpass study, and data from 66 cell lines from Keats lab. Transcriptomic correlation analysis was used to compare the data from cell lines with patient-derived primary myeloma cells. Created in BioRender.com.

In this study, RNA and protein surface expression levels of relevant drug-related targets, including molecules relevant for immunotherapy, were analyzed using flow cytometry and RNA sequencing (RNAseq). We examined a set of non-hyperdiploid and hyperdiploid in-house cell lines, as well as some well-established cell lines. Further, transcriptomic correlation analysis was used to compare cell lines with primary MM cells, to study how well cell lines resembled patient myeloma disease (schematic overview in Fig. 1). Lastly, we describe three previously uncharacterized myeloma cell lines, IH-1, URVIN and FOLE, the two latter being novel cell lines.

## Results

### Expression of cell surface markers in HMCLs

**Fig. 2:**
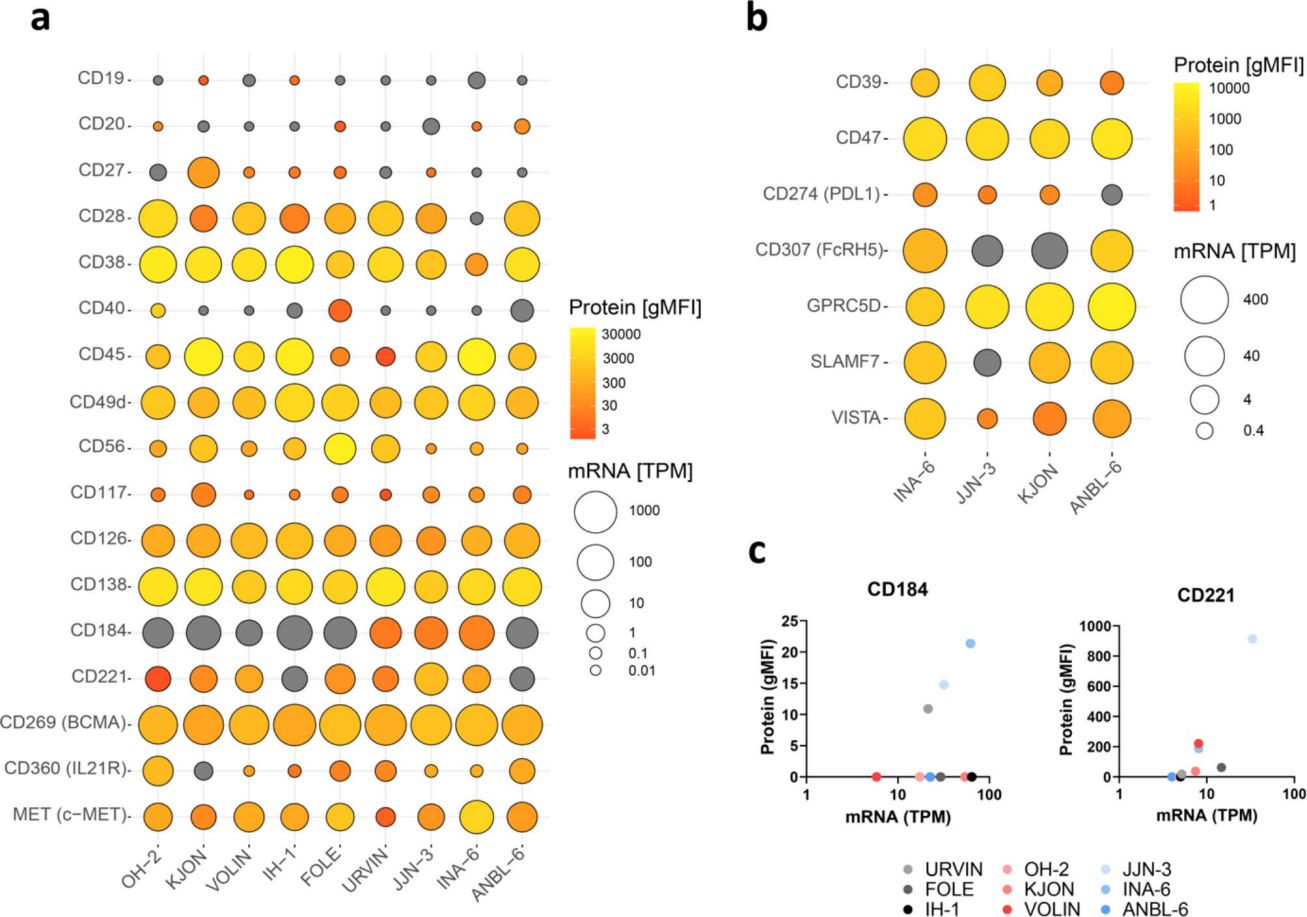
RNA and protein expression levels of a variety of markers in nine different human myeloma cell lines. (**A**,**B**) Protein expression levels were analyzed using flow cytometry (see Supplementary Table 1) and given as geometric mean fluorescence intensity (gMFI) levels. The percentage of cells expressing these markers is shown in Supplementary Fig. 1. mRNA expression levels are based on RNAseq data. (**C**) Correlation plots of gMFI and mRNA expression for the surface proteins CD184 and CD221. Hyperdiploid cell lines are shown in red colors, while non-hyperdiploid cell lines are shown in grey (in-house) and blue colors. TPM: transcripts per million. Grey color indicates gMFI = 0. Scales for both gMFI and TPM are logarithmic.

Protein expression of cell surface markers in nine HMCLs was analyzed using flow cytometry. To evaluate how well the protein expression of surface markers correlated with transcript levels, RNAseq analysis of these cell lines was also performed. Six of the HMCLs were in-house, and included three hyperdiploid cell lines, OH-2^3,4^, VOLIN and KJON^5^, and the three previously uncharacterized non-hyperdiploid cell lines, IH-1^6^, URVIN and FOLE. In addition, we included the well-established non-hyperdiploid cell lines INA-6, ANBL-6 and JJN-3. We first tested some markers that are commonly used to characterize myeloma cell clones, including markers that could distinguish between true HMCLs and Epstein-Barr virus positive lymphoblastoid cell lines, such as CD19, CD20 and CD117^7^. Certain markers, such as CD38, CD138 (*SDC1*), CD269 (BCMA, *TNFRSF17*) and CD49d (*ITGA4*) are characteristic of MM primary cells and HMCLs, making them potential targets for therapy^8^. Although there was some variation in the mRNA expression of these markers, the cell surface protein expression was high in all the cell lines (Fig. 2A, Supplementary Fig. 1A, and Supplementary Tables 2 and 3), except for INA-6, which had low levels of CD38 (gMFI 78.33 versus median gMFI 9611.35; TPM 2.59 versus median TPM 55.22, Supplementary Table 2), consistent with previous studies^9^. Interestingly, for many of the other surface markers, we found large variation in expression levels between the different cell lines. For instance, the growth factor receptors CD221 (*IGF1R*) and CD360 (*IL21R*) varied from undetectable to clearly expressed among the cell lines (CD221: gMFI 0–913.95; TPM 4–33.2, CD360: gMFI 0–699.07; TPM 0.03–15.58). Another example is CD27, which is heterogeneously expressed in patient myeloma cells and where low levels have been found to correlate with poor prognosis^10^. In cell lines, this marker is often lacking^10^, but here we could detect both CD27 mRNA and protein in the hyperdiploid cell line KJON (gMFI 105.4; TPM 20.89)(Fig. 2A).

### Expression of immunotherapy relevant targets

We went on to study additional relevant myeloma drug targets and markers relevant for immunotherapy. As efficacy of immunotherapy will depend on cell surface protein expression of target protein^11,12^, we analyzed a set of established and emerging immunotherapy-relevant targets in a subset of the cell lines; INA-6, JJN-3, KJON and ANBL-6. For GPRC5D, a target for the bispecific antibody talquetamab^13^, both mRNA and surface protein were expressed in all the tested cell lines (median gMFI 3800.5; median TPM 234.92) (Fig. 2B, Supplementary Fig. 1B, and Supplementary Tables 4 and 5). CD307 (*FCRH5*), targeted by the bispecific antibody cevostamab, showed a huge variation in expression levels between the different cell lines (gMFI 0–974; TPM 6.1–128.93), and had very low expression in JJN-3 (gMFI 0; TPM 6.1) and KJON (gMFI 0; TPM 16.75). SLAMF7 (*SLAMF7*)^13^, targeted by the monoclonal antibody elotuzumab, also showed huge variation between the cell lines (gMFI 0–655; TPM 3.08–82.63), and had low expression in JJN-3 (gMFI 0; TPM 3.08). We also tested some other establised and less established therapy-related targets. Specifically, PDL1^14^, a protein involved in immune evasion, showed low or no expression in all four cell lines (median gMFI 14.45; median TPM 0.86). For CD39 (*ENTPD1*), an important enzyme for conversion of ATP to immunosuppressive adenosine^15^, and the checkpoint receptor CD47^16^, both mRNA and surface protein were expressed in all the tested cell lines (CD39: median gMFI 361.95; median TPM 3.02, CD47: median gMFI 2346; median TPM 66.98). VISTA (*VSIR*)^17^, a checkpoint receptor, found to be a prognostic factor as high expression is linked to poor prognosis^17^, had its highest expression in INA-6 (gMFI 786; TPM 52.72).

### Correlation between transcript and cell surface protein expression levels

Cell surface protein expression and transcript levels of the different markers correlated well between cell lines, with median correlation (R^2^) between cell surface protein expression (gMFI) and transcript (TPM) levels being 0.81 (markers tested in all nine cell lines; Fig. 2A) and 0.64 (markers tested in a subset of cell lines; Fig. 2B) (Supplementary Tables 2 and 4). However, some markers did not correlate that well. For instance, CD184 (*CXCR4*) mRNA was expressed in all cell lines (median TPM 29.28), yet expression of surface protein was absent in six of the nine cell lines (median gMFI 0). CD221 (*IGF1R*) mRNA was also expressed in all cell lines (median TPM 7.43), but protein expression was low/absent on the surface of OH-2, IH-1 and ANBL-6 (gMFI 0; median gMFI 37.75) (Fig. 2C). These data further suggest that surface expression of proteins should be investigated when considering using HMCLs to model MM disease.

### Transcriptome correlation analysis of cell lines and patient cells

**Fig. 3:**
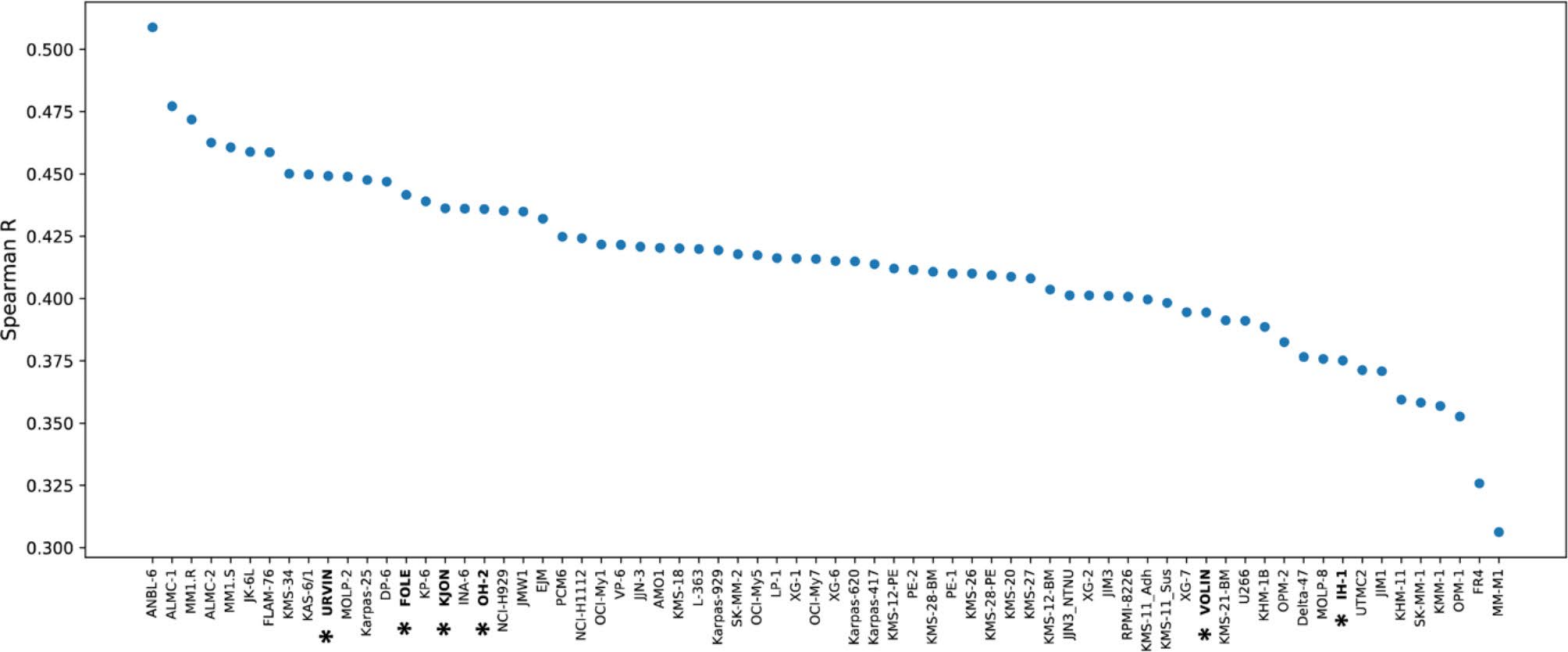
Correlation of transcriptomic data from human myeloma cell lines and myeloma patients.A comparison of transcriptomic data from 66 cell lines from Keats lab (www.keatslab.org) and 6 in-house cell lines (marked with *), correlated to 692 MM patient samples from the MMRF CoMMpass study IA16, based on RNAseq data normalized via variance stabilizing transformation. The comparison was done similarly as described in Sarin et al^18^.

An earlier study investigated how well cell lines resembled patient myeloma cells^18^, by comparing a panel of 66 HMCLs (www.keatslab.org) to 779 newly diagnosed MM (NDMM) patient samples from the MMRF CoMMpass study^19^. We wanted to extend this analysis with our in-house cell lines, including the three hyperdiploid cell lines. We therefore reanalyzed the data, and as controls we included our own clones of the cell lines ANBL-6, OH-2, INA-6, and JJN-3, which were also part of the original study. The transcriptomes were compared based on global gene expression patterns to determine how similar each cell line was to patient cells. ANBL-6 is the cell line that resembles patient samples best based on these datasets^18^, and the ANBL-6 clone in our laboratory showed a similar correlation, and together with OH-2 and INA-6 mirrored the previous study’s findings. JJN-3 showed a rank deviation (Supplementary Fig. 2A), possibly because we used a different clone of JJN-3 than the one used by Keats lab (see Methods and Discussion). Notably, four of our six in-house cell lines, FOLE, KJON, OH-2, and URVIN, fall into the category of cell lines that represent patient disease well, defined as all ranking among the top 20 cell lines (Fig. 3).

It was of further interest to see whether our hyperdiploid cell lines resemble patients with hyperdiploid disease better than non-hyperdiploid cell lines do. Given the scarcity of hyperdiploid cell lines, there is a lack of studies that have evaluated their usefulness to model patient disease. In the original study from Sarin et al.^18^, only the OH-2 and KP-6 hyperdiploid cell lines were included in the analysis. We divided the MM patients into a hyperdiploid (*n* = 395) and non-hyperdiploid (*n*= 297) group. Surprisingly, many cell lines resembled both the hyperdiploid and the non-hyperdiploid samples, with ANBL-6 resembling both hyperdiploid and non-hyperdiploid disease more than any other cell line (Supplementary Fig. 2B, C). However, the hyperdiploid cell lines were more similar to hyperdiploid patients than to non-hyperdiploid patients, and the hyperdiploid cell lines VOLIN and KJON were among the five cell lines that had the largest difference in their correlation between hyperdiploid and non-hyperdiploid patient samples (Supplementary Fig. 2D). These results support that inclusion of hyperdiploid cell lines adds value to in vitro studies of MM^20^.

### Characterization of three novel myeloma cell lines

Lastly, we here present three previously uncharacterized cell lines, FOLE, URVIN, and IH-1^6^, established in our lab, showing results from karyotyping, fluorescence in situ hybridization (FISH), fingerprinting and patient characteristics^21^(Supplementary Fig. 3). All three cell lines had a t(4;14) and a non-hyperdiploid karyotype (full karyotypes found in Supplementary Fig. 3A). IH-1 was published earlier, but not genetically characterized^6^. Our present analysis revealed that it has a del13 and 1q amplification. URVIN was derived from peripheral blood of a terminally ill 52-year-old male myeloma patient with secondary plasma cell leukemia, and the clinical parameters measured at sampling were albumin: 32 g/L, and IgG-kappa: 2.8 g/L. URVIN also harbored a del1p, 1q amplification, del13 and del17. FOLE was derived from the peripheral blood of a 68-year-old male and found to also harbor a del1p, 1q amplification, del13 and del17. Clinical parameters measured at sampling were albumin: 70 mg/L, IgA: 1 g/L, and free lambda light chains: 932 mg/L. Characterization of the novel cell lines and the other included cell lines are shown in Table 1. FOLE and URVIN resembled myeloma patient cells well based on transcriptomic correlation, ranking among the top 15 cell lines, and IH-1 to a lesser extent, ranking 61/70 (Fig. 3).

**Table 1:** Cell line characteristics.

Cell line		#Chr.	Ploidity	del17	TP53	del13	1p	1q	IL-6 dependent	References
In- house	URVIN	45	NHRD, t(4;14)	del17	ND	del13	del1p	gain1q	Yes	
	FOLE	37–77	NHRD, t(4;14)	del17	C238W	del13	del1p	gain1q	No	
	IH-1	51–56	NHRD, t(4;14)	normal	WT	del13	normal	gain1q	Yes	^6^
	KJON	47–48	HRD, + 9,15,19	del17	Y234*	del13	del1p	gain1q	Yes	^5^
	VOLIN	76	HRD, + 3,5,7,15,19,21	normal	L130V	normal	normal	gain1q	No	^5^
	OH-2	48–51	HRD, + 3,7,15,19,21	normal	WT	del13	del1p	gain1q	Yes	^3,4^
Commercial	INA-6	82	NHRD, t(11;14)	normal	Q331X	normal	normal^#^	normal^#^	Yes	^33^
	ANBL-6	82	NHRD, t(14;16)	del17	WT	del13	del1p^#^	gain1q^#^	Yes	^32^
	JJN-3	60	NHRD, t(4;14), t(14;16)	del17	Missing	del13	gain1p^#^	gain1q^#^	No	^34^

HRD: hyperdiploid; NHRD: non-hyperdiploid # Chromosome Genomic Hybridization array of cell lines, data not shown.

## Discussion

In this study we compared MM cell lines with respect to their resemblance to primary tumour cells on the transcript level. We also directly compared a selection of cell surface markers with mRNA levels in a panel of cell lines. Some cell surface markers are important to determine patient prognosis whereas others are direct targets for immunotherapy. For instance, patients with high basal expression levels of CD38 have been shown to respond better to daratumumab than those with lower expression levels^11^, highlighting the clinical importance of CD38 expression levels. Previous studies have demonstrated that CD38 is expressed in all MM cells from patient samples, both at the RNA^22,23^and protein^11,22^levels. However, significant heterogeneity in CD38 cell-surface expression intensity has been observed^11^. In our study, we also observed variability in CD38 expression. While all cell lines express the CD38 transcript (TPM > 1), INA-6 exhibits notably lower transcript levels (TPM 2.59 versus median TPM 55.22), and reduced cell surface expression compared to the other cell lines (gMFI 78 versus median gMFI 9611.35, Supplementary Table 2). This variability in CD38 expression highlights the importance of selecting cell lines with appropriate CD38 levels for studies targeting CD38-mediated therapies, as expression levels may significantly impact therapeutic response.

Another emerging target for immunotherapy in MM is GPRC5D, targeted by the bispecific antibody talquetamab^24^. A significant positive association between GPRC5D expression on MM cells and the efficacy of talquetamab has been reported, along with significant heterogeneity in MM cell surface expression of GPRC5D among MM patients^12^. In the present study, all tested cell lines exhibited high levels of GPRC5D cell surface expression. Therapies targeting BCMA, such as CAR T cell therapies (e.g., idecabtagene vicleucel and ciltacabtagene autoleucel) and BCMA-directed bispecific antibodies like teclistamab, have shown substantial efficacy in the clinic^25^. In the present study, all analyzed cell lines expressed BCMA at both the RNA and protein levels, consistent with previous reports on MM cells from patient samples^23,26^.

For some genes, the transcript levels did not correlate well with protein levels. For instance, we clearly detected CD184 (*CXCR4*) transcript in all the cell lines, whereas CD184 cell surface expression (gMFI) only was detected in three out of the nine cell lines (URVIN, JJN-3, and INA-6). SLAMF7 was expressed at the transcript levels in all cell lines, consistent with previous findings^23^; however, no surface protein expression was detected in JJN-3. Although RNA expression was low (TPM 3) it was above our cut-off (TPM > 1). Discrepancies between RNA and surface protein expression may reflect variations in receptor turnover in the analyzed cell lines, potentially due to downregulation following binding of their specific ligands^8,27^, or post-transcriptional regulation^28^.

In an earlier study by Sarin et al.^18^, transcriptional correlation profiling between cell lines and patient samples revealed that some cell lines, particularly ANBL-6, more closely represented patient disease, while others – despite their frequent use – scored relatively poorly in their similarity to patient tumours. Importantly, none of the MM cell lines fully replicated patient disease, with median R values ranging from 0.35 to 0.54 (i.e., far from 1)^18^. However, these findings highlight that, while no MM cell line is perfect, some seem to better represent patient disease than others. In the present work, we applied the same correlation assay but included additional transcriptomic data from our in-house cell lines. Consistent with the previous study, ANBL-6 emerged as the cell line most representative of patient disease, while FR4 and MM-MM.1 showed the lowest correlation.

We also included four cell lines cultivated in our lab, ANBL-6, OH-2, INA-6, and JJN3, which were part of the earlier study. As expected, the cell lines mostly mirrored the findings of the original study. One exception was JJN-3 which showed some deviations, likely because we used a different clone of JJN-3 than the one used by the Keats lab (see Methods). For instance, our JJN-3 clone carries an *NRAS *mutation^29^, which has also been identified by other users than us. This mutation, due to its one-in-three ratio of mutated alleles, may result in small peaks in sequencing data and could have been overlooked in some studies^30^. Among our six in-house cell lines, four (URVIN, FOLE, KJON, and OH-2) showed strong resemblance to patient disease, ranking in the top 20 of 70 cell lines. Conversely, VOLIN and IH-1 ranked in the bottom 10, showing weaker correlation.

When splitting the patient samples into hyperdiploid and non-hyperdiploid groups, we expected the hyperdiploid cell lines to rank higher. However, differences were minor, and the cell lines that best resembled all patients regardless of group also best resembled hyperdiploid patients. Still, the hyperdiploid cell lines showed the most significant ranking improvement for hyperdiploid patients: KJON improved from 24 to 13, KP-6 from 20 to 12, and VOLIN from 59 to 49, while OH-2 remained unchanged at 17. Surprisingly, 12 non-hyperdiploid cell lines still correlated better with hyperdiploid patients than the highest ranking hyperdiploid cell line. This unexpected result could either be due to the fact that we had a very limited number of hyperdiploid cell lines in the analysis, or it could suggest that inclusion of hyperdiploid cell lines in studies is not absolutely required. MM cell lines generally resemble more aggressive, poor-prognosis disease states rather than “typical” newly diagnosed MM. Fast-growing tumour cells express high levels of transcripts important for proliferation and cell division, which may influence these rankings. Some of our in-house cell lines, such as OH-2, URVIN and KJON, may better correlate with low-risk NDMM, given their dependence on IL-6 and other supplements, similar to NDMM cells reliant on the bone marrow microenvironment. KJON also expressed CD27, a marker that is expressed in low levels in patients with high-risk NDMM, further supporting that KJON could represent low-risk NDMM well^31^. Of note, in the Keats dataset only three other cell lines (KMS12BM, MOLP2, and XG6) expressed CD27 mRNA. Additionally, since these rankings are based on transcript levels, protein expression should also be considered when selecting cell lines for studies.

In summary, analysis of RNA and protein expression levels of relevant drug-related targets shows a good correlation between the mRNA level and the surface protein level, although exceptions were observed. When studying MM using cell lines, we suggest including cell lines that cover the range of expression of the protein (or gene) of interest. Importantly, since expression of many markers varied between cell lines they should be selected carefully. Further, using transcriptomic correlation analysis to compare HMCLs with primary MM cells, we show that hyperdiploid cell lines are good models for myeloma disease, and represent hyperdiploid disease better than non-hyperdiploid disease.

## Methods

### Cell lines

ANBL-6, INA-6, and JJN-3 were kind gifts from Dr. D. Jelinek (Mayo Clinic, Rochester, MN, USA), Dr. M. Gramatzki (University of Erlangen-Nurnberg, Erlangen, Germany), and Dr. J. Ball (University of Birmingham, UK), respectively^32–34^. Of note, the Keats Lab JJN-3 clone was from Leibniz Institute DSMZ and differs from the clone from Dr. Ball both in terms of fingerprinting (Supplementary Fig. 3), expression of genes and the lack of an *NRAS *mutation. The other 6 cell lines were established in our laboratory. Of these, KJON, OH-2 and VOLIN have been karyotyped before^4,5^, whereas IH-1^6^, FOLE and URVIN are characterized for the first time in this paper. All cell lines except FOLE and ANBL-6 were kinome sequenced previously^29^. All in-house cell lines have been tested and found negative for Epstein-Barr virus (Department of Microbiology, St. Olav’s Hospital), and all cell lines are regularly tested for mycoplasma negativity (MycoAlert Plus, Lonza Ltd., Basel, Switzerland). All cell lines were cultivated in cell culture flasks (Corning, Sigma Aldrich) at 37 °C and 5% CO_2_. Growth medium was RPMI with either fetal calf serum (FCS) (Gibco, #10270106) or human serum (HS) (Department of Immunology and Transfusion Medicine, St. Olav’s Hospital, Trondheim, Norway). Some cell lines were supplemented with 1 ng/mL IL-6 (Gibco, #PHC0065) or IL-6 supernatant (sup.). IL-6 sup. was isolated from peripheral blood leukocytes from buffy coat after culturing 24 h with 0.1 µg/mL lipopolysaccharide (Sigma Aldrich, #L4391), yielding a supernatant activity comparable to 2 ng/mL IL-6. URVIN was cultivated in 10% FCS in RPMI with IL-6 sup., FOLE in 10% HS in RPMI, IH-1, OH-2, and KJON in 10% HS in RPMI with IL-6 sup., VOLIN and JJN-3 in 10% FCS in RPMI, INA-6 and ANBL-6 in 10% FCS in RPMI with IL-6. In-house cell lines are available upon request. 

### Flow cytometry

250 000 cells per sample (exception: for GPRC5D, 500 000 cells per sample were stained) were incubated with antibody or isotype control in a volume of 50 µL for 30 min, washed, and analyzed using an LSRII flow cytometer (BD Biosciences, San Jose, CA, USA). A three-coloring technique was used with fluorochromes FITC, PE, and APC in each flow tube, and fluorescence compensation was performed using OneComp eBeads Compensation Beads (Invitrogen, #01–1111-41). For a selected set of immunotherapy targets, flow cytometry was performed individually, without compensation. Viable cells were gated on FSC/SSC. For all the markers, the gates for positive and negative cells were set using isotype controls. Details about all antibodies used can be found in Supplementary Table 1. Data were analyzed with FlowJo software V.10 (BD Biosciences, San Jose, CA, USA).

### RNA sequencing

RNAseq of the in-house cell lines (NTNU) was performed using Lexogen SENSE mRNA library kit with 500 ng input RNA and sequenced on a NextSeq HO flowcell with 2 × 75 bp PE reads, giving an average output of ~ 28.3 M reads/sample (range 19.7–42.3). Two biological replicas were included for each cell line. Output was averaged for each cell line and used with cell line data from Keats Lab and patient data from the MMRF CoMMpass study IA16. For samples from the CoMMpass study, these were RNAseq analysis on CD138 enriched cells with purity > 80%^19^. This procedure follows supplementary methods from Sarin et al.^18^. Raw fastq sequences were aligned with Hisat2 using GRCh37 (hg19) genome and transcriptome, using the Hisat2 pre-indexed hg37_tran transcriptome/genome. Gene mapping was performed by the program featureCounts (Subread package), with multimapping reads counted (-M), reads assigned to all overlapping meta-features, assigning fractional counts (--fraction), and with transcriptome annotation Homo_sapiens.GRCh37.75.gtf (-a). We mapped 63677 genes in the 18 cell line samples from NTNU, where 48637 genes overlapped between NTNU, CoMMpass (943 samples) and the HMCL66 (66 samples) data. We then created two new data tables, one consisting of the samples from CoMMpass and HMCL66 (CH table – 48638 genes and 1000 samples), and one consisting of the samples from CoMMpass, HMCL66 and NTNU (CHM table – 48637 genes and 1020 samples). Note that all the shared genes between CoMMpass and HMCL66 were also present in NTNU. The expression values in the combined table were converted to cpm (counts per million), and genes were further filtered as described in Sarin et al.^18^ (cpm > 1 in 2 or more samples). This resulted in 23773 genes after filtering. A variance stabilizing transformation was first performed on the CH data table separately using the varianceStabilizingTransformation (blind = TRUE, fitType=’parametric’) function from the deseq2 package in R. We then used IQR (Interquartile range) to identify the 5000 most variable genes in the CH data table. We then performed the same variance stabilizing transformation on the CHM data table, and then calculated Spearman Rank correlations using the CHM data table. This was done to focus the correlation towards the 5000 genes used in the original analysis by Sarin et al.^18^. Each of HMCL66 and NTNU cell lines were correlated to each of the CoMMpass samples, creating a correlation table with 943 correlation values for each cell line. The cell lines COLO677_DSMZ_p8 (genetically identical to RPMI-8226) and Karpas929_ECACC_p15 (a different passage included) from HMCL66 were removed. Correlation profiles from the replicate cell lines from NTNU (two for each cell line) were averaged, and the average correlation for the cell lines IH-1, JJN3_NTNU, KJON, URVIN, VOLIN, FOLE were used for comparison. ANBL-6, INA-6, and OH-2 were included as controls. Supplementary Fig. 2A shows averages for all NTNU cell lines. (Fig. 3 in the paper: data for ANBL-6, OH-2, and INA-6 are from HMCL66/Keats Lab, the supplementary figure displays averages for all NTNU cell lines). From the table “MMRF_CoMMpass_IA16a_CNA_LongInsert_FISH_CN_All_Specimens” including 1077 samples with hyperdiploid status, we selected 780 samples with extension “1_BM” (_1 = Newly diagnosed MM). Of these, 692 overlapped with the CoMMpass gene expression data, of which 395 had hyperdiploid (HRD1) status and 297 had non-hyperdiploid (HRD0) status. We calculated average correlations between cell lines for (1) All samples in CoMMpass (692) (2) All samples with HRD1 status (395), and (3) All samples with HRD0 status (297).

### G-banding and fluorescence in situ hybridization (FISH) analyses

Cells were short-term cultured, harvested, stained for G-banding analysis, and analyzed cytogenetically as previously described^35,36^. The karyotypic description followed the recommendations of the International System of Cytogenomic Nomenclature^37^. FISH was performed on interphase nuclei and metaphase spreads using the IGH/FGFR3 dual fusion probe (Cytocell, Oxford Gene Technology, Begbroke, Oxfordshire, UK) and the CKS1B/CDKN2C amplification/deletion probe (Cytocell) according to the manufacturer’s instructions. Chromosome preparations were counterstained with 0.2 µg/ml 4’,6-diamidino-2-phenylindole and overlaid with a 24 × 50 mm^2^ coverslip. Fluorescent signals were captured and analyzed using the CytoVision system (Leica Biosystems, Newcastle, UK).

## Electronic supplementary material

Below is the link to the electronic supplementary material.

Supplementary Figures

Supplementary Tables

## Data Availability

Processed data from RNA sequencing can be found in GEO under accession number GSE250236. Access to raw data or in-house scripts used in the methods will be provided on request by contacting the authors.
