# Supplementary material for: Cell surface marker heterogeneity in human myeloma cell lines for modeling of disease and therapy
Source: Sci Rep. 2024 Nov 20;14:28805. doi: 10.1038/s41598-024-80263-y (PMC11579509; doi:10.1038/s41598-024-80263-y)
Supplement: Supplementary file 1 — Supplementary Figures [file 41598_2024_80263_MOESM1_ESM.pdf]

Supplementary Figures

Supplementary Figure 1

(A) (B)

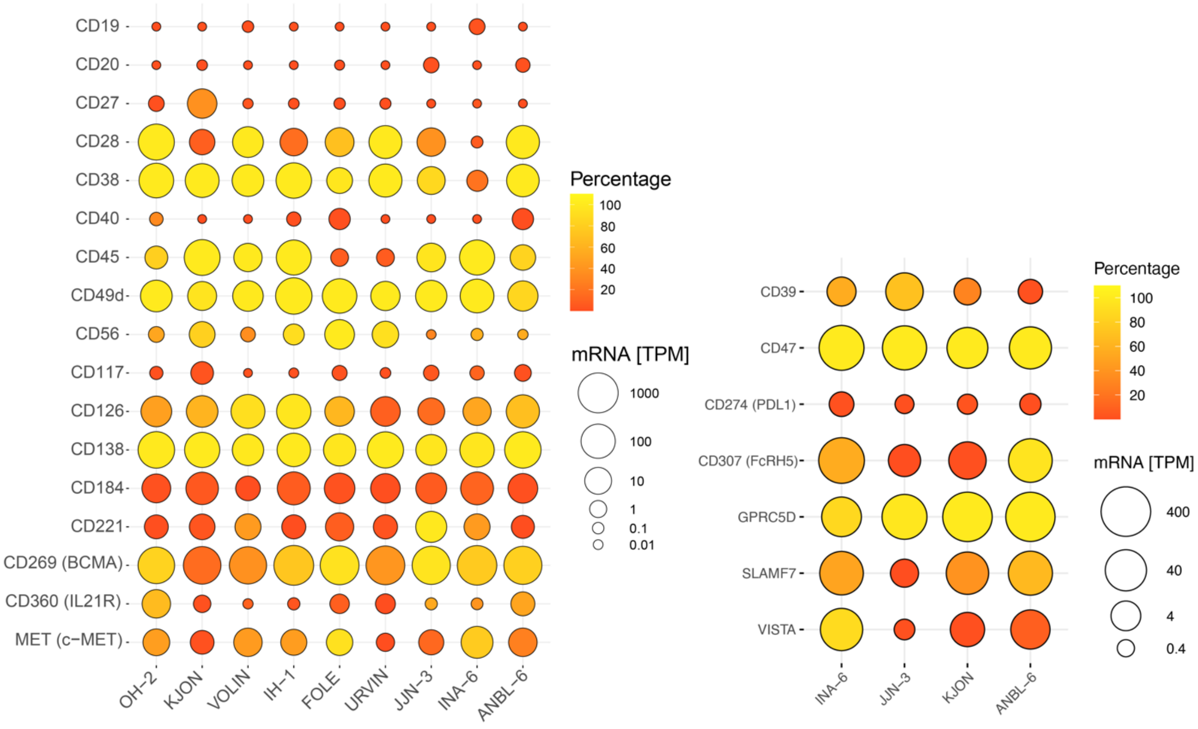

Supplementary Figure 1

(A) Protein expression levels were analyzed using flow cytometry and are shown as % positive cells. The gMFI levels of these markers are given in Figure 2A. The mRNA expression levels are based on RNAseq data and are the same as in Figure 2A. (B) Selected set of immunotherapy target genes in a subset of cell lines. TPM: transcript per million. Scales for percentage and TPM are linear and logarithmic, respectively.

Supplementary Figure 2

(A)

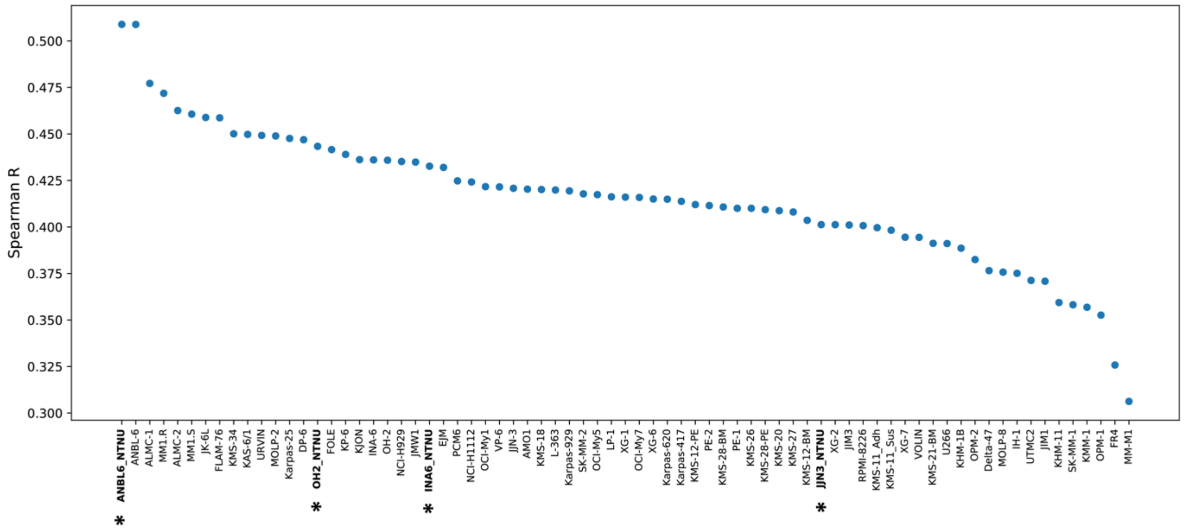

(B)

Hyperdiploid:

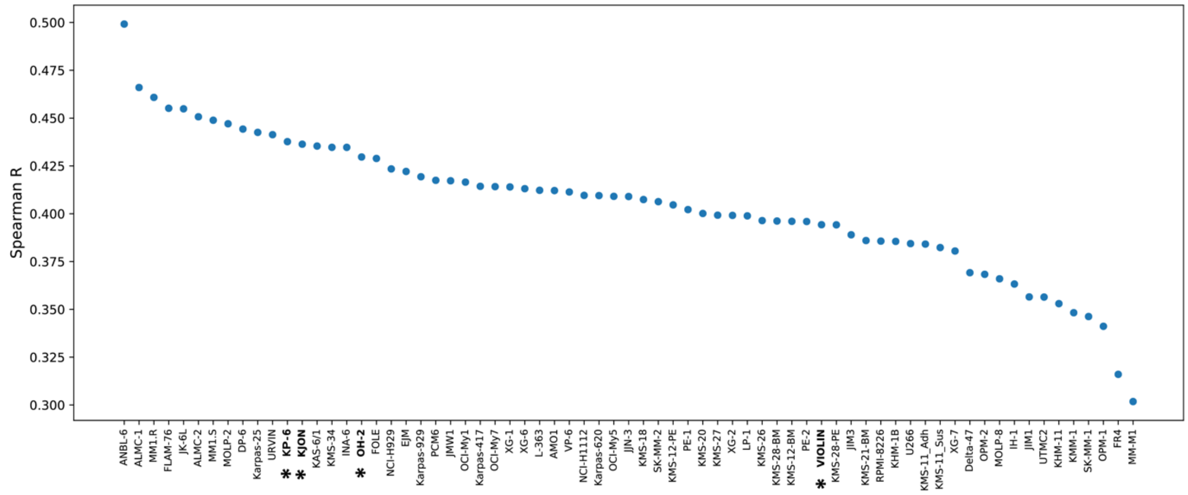

(C)

Non-hyperdiploid:

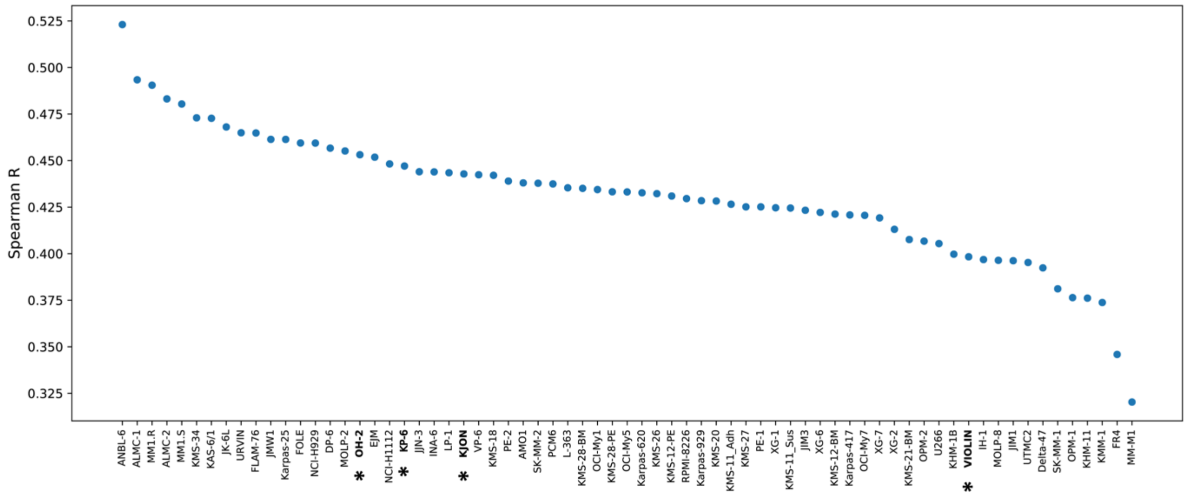

(D)

Difference non-hyperdiploid – hyperdiploid:

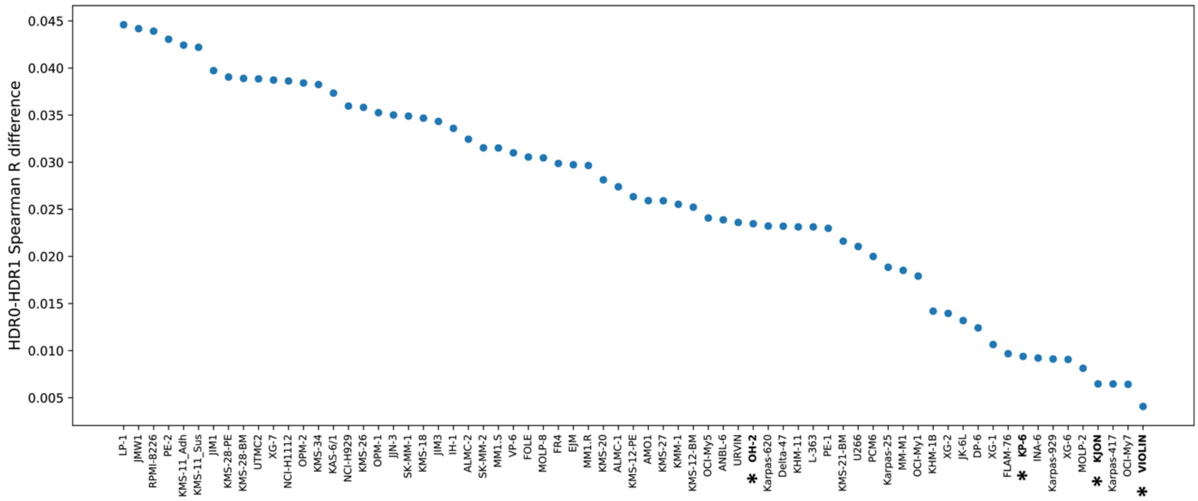

Supplementary Figure 2

(A) Correlation between transcriptomes of myeloma cell lines, from Keats lab (www.keatslab.org) and our in-house cell lines, and transcriptomes of patient MM cell samples from the MMRF CoMMpass study[1]. “Control cell lines” included from our lab shown in bold and marked with *. (B,C) A comparison of available cell lines from Keats lab and our in-house cell lines (hyperdiploid cell lines marked with *) correlated to hyperdiploid (B) and non-hyperdiploid (C) patient MM cells, using RNAseq data normalized via variance stabilizing transformation. (D) The difference between the cell lines’ correlation to hyperdiploid MM patients versus their correlation to the non-hyperdiploid patients. Cell lines more similar to the hyperdiploid patients are shown to the right (hyperdiploid cell lines marked with *).

Supplementary Figure 3

A–I: IH-1

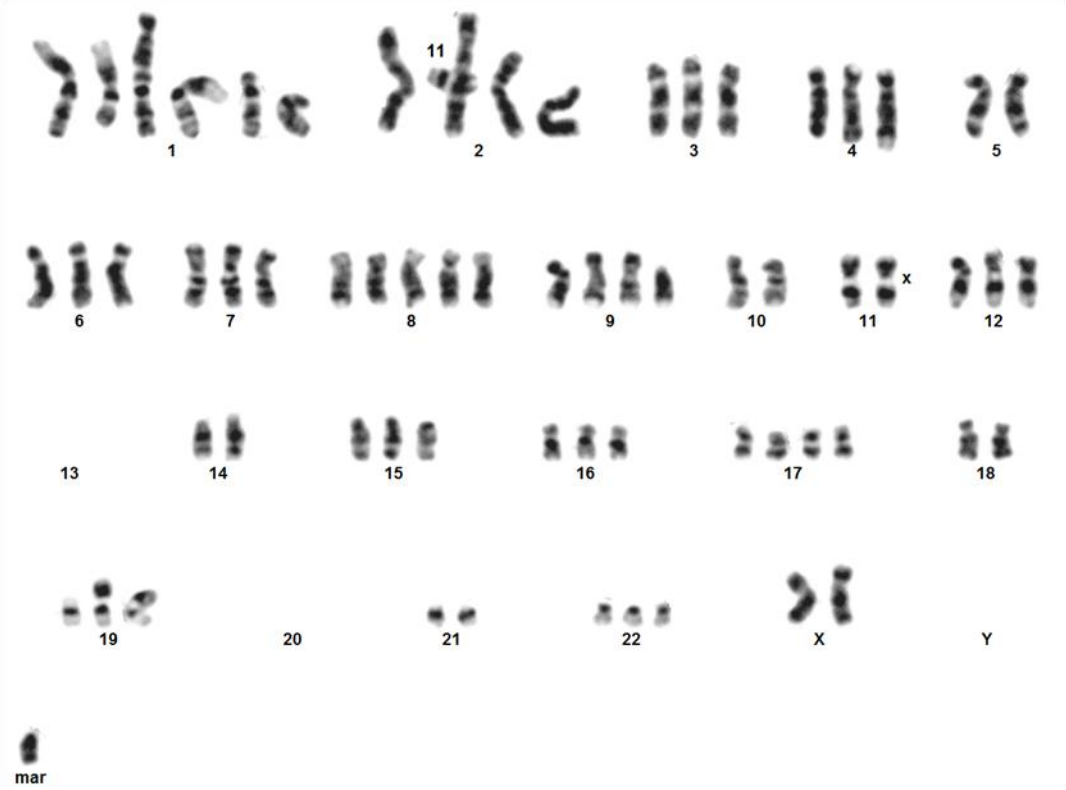

51~66<3n>,XX,-Y,dic(1;5)(p11;q33),+add(1)(p11)x2,+add(1)(q23),+2, der(2)t(2;13)(p23;q11)x2,der(4)t(1;4)(p36;q35), der(4)t(4;14)(p16;q32)x2, +8,add(8)(p11)x2, +del(9)(p11),-10,-13,-13,-13,-14,-14, der(14)t(4;14)(p16;q32),+17, -18, der(19)t(11;19)(q15;p13)x2,-20,-20,-20,-21[cp12]

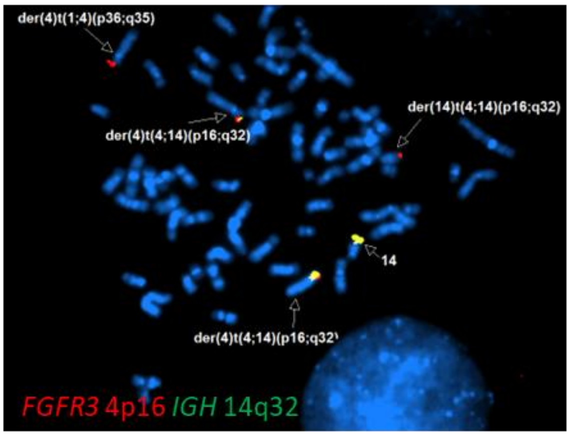

A-II: URVIN

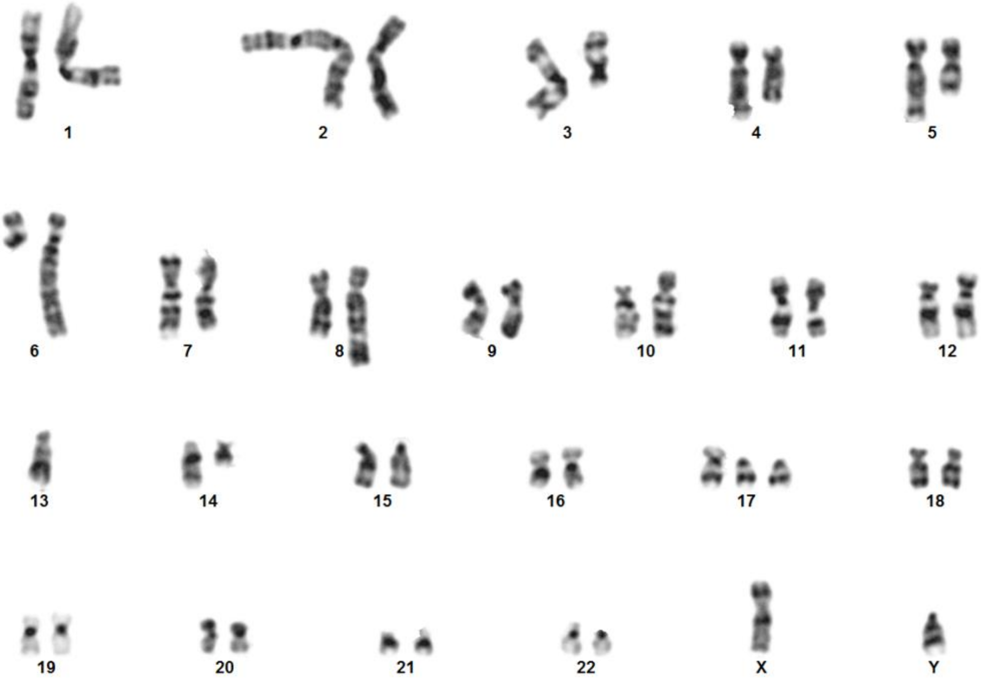

45,XY,der(1)del(1)(p32p34)inv(1)(p12p22),der(2)t(1;2)(q11;p25)del(2)(q24q31), t(3;8)(q13;q24),der(4)del(4)(p11p15)del(4)(q21q27)t(4;14)(p16;q32),del(5)(q11q22),t(6;6)(q13; q25), t(7;9)(q32;q22),del(10)(p11),del(11)(q13q15),del(12)(p12p13),-13,-14, der(14)t(4;14)(p16;q32),der(14)del(14)(q13q31)t(4;14)(p14;q32), del(17)(p11)+21[5]/46,idem,+del(17)(p11)[6]/46,idem,t(Y;9)(q11;q22),+del(17)(p11)[4]

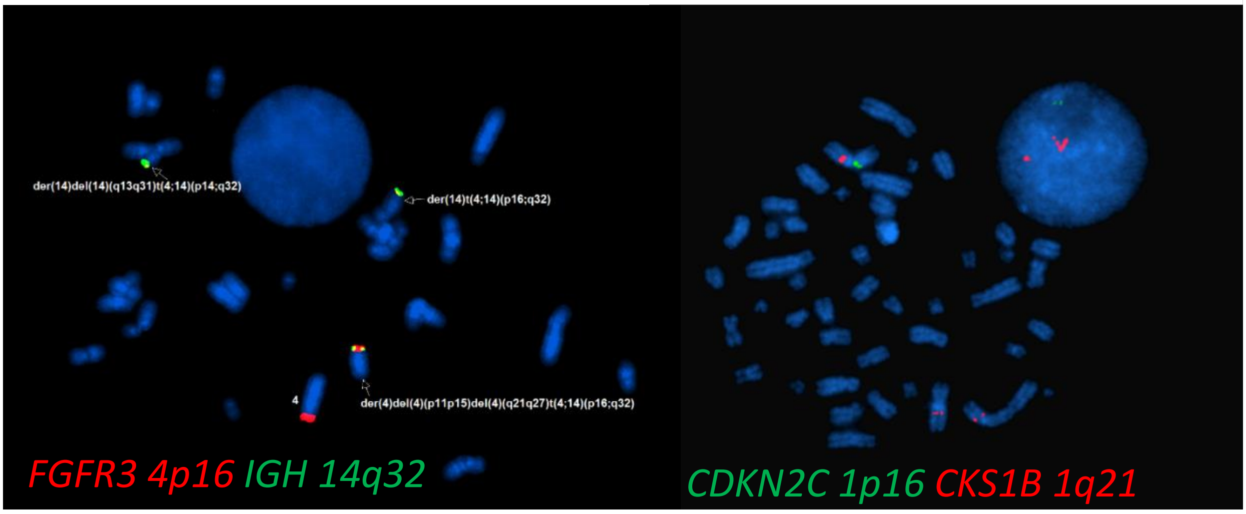

A-III: FOLE

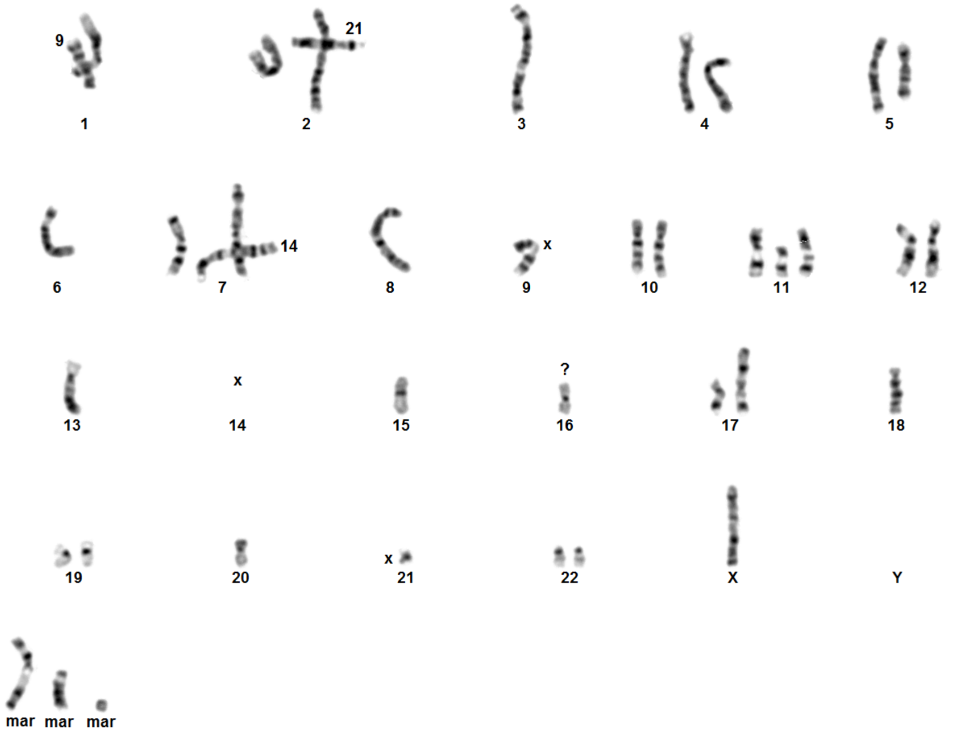

37~39,add(X)(p11,-Y,add(1)(q21),t(2;8)(p?13;q24),-3,der(1)t(1)(p11;q27), der(4)t(4;14)(p16;q32),
del(5)(q13q22),-6,add(7)(p13),-8, der(11)del(11)(p11)del(11)(q15q23),
+der(11)(pter→p11::q25→q23::q13→p11::q13→q23::q25→qter),add(12)(p11),-13, ?add(13)(p11),-
14,der(14)t(1;14)(q12;q32),-15,-16,add(17)(p13),-18, der(18)t(1;18)(q25;q21),-20,del(21)(q21),
der(21)(21pter→21q22::?::3p11→3pter),+3mar [cp2]

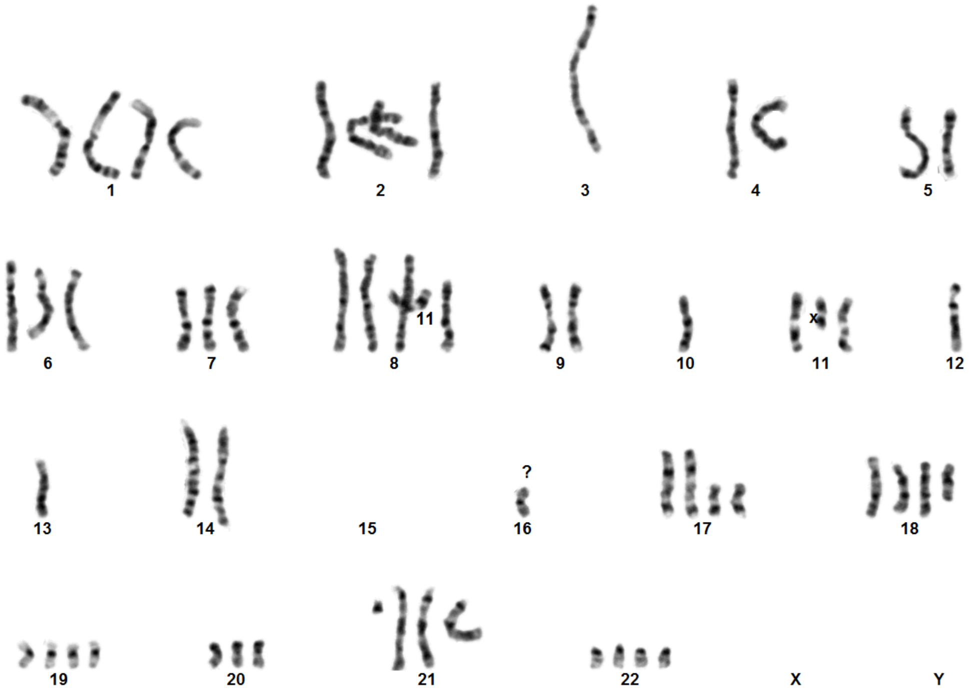

47~77,add(X)(p11), -X,-Y,-Y,add(1)(p12)x2,add(1)(q21)x2,t(2;8)(p?13;q24)x2,-3,-3,der(1)t(1)(p11;q27)x2,-4,-5,
del(5)(q13q22)x2,der(6)del(6)(p23)add(6)(q23)x2,-7,t(7;9)(p13;q21),-10,-
10,der(11)del(11)(p11)del(11)(q15q23)x2, +der(11)(pter→p11::q25→q23::q13→p11::q13→q23::q25→qter)x2,-
12,add(12)(p11)x2, -13,-13, ?add(13)(p11)x2,-14,-14,der(14)(t(1;14)(q12;q32)x2,-15,-15,-16,-16,-16,-17,
add(17)(p13),-18, der(18)t(1;18)(q25;q21)x2,-19,-20,-20,del(21)(q21)x2,
der(21)(21pter→21q22::?::3p11→3pter)x2, 1~5mar[cp11]

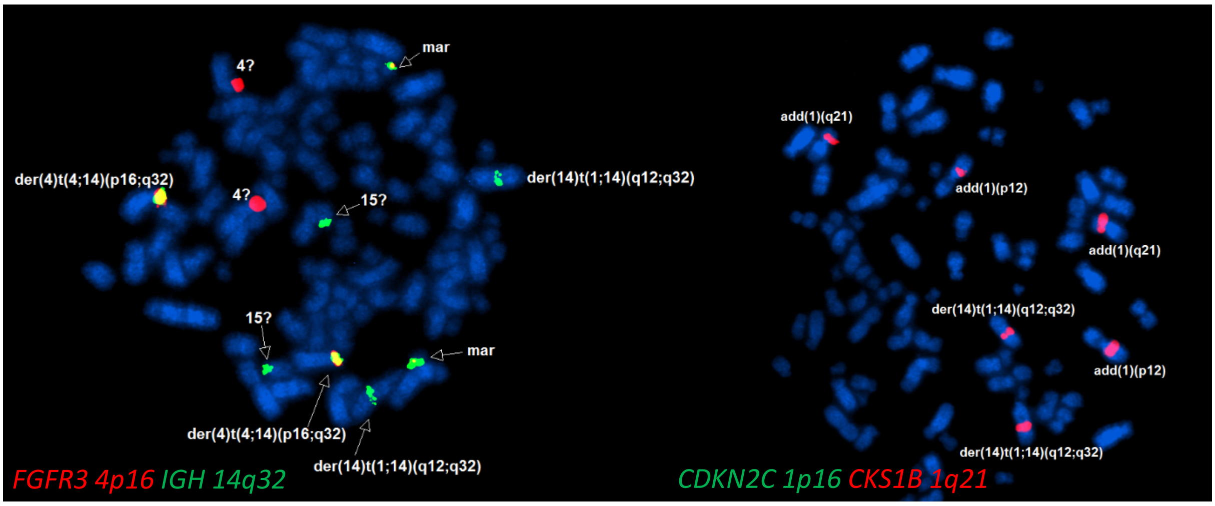

B.

Cell line	Fingerprint score	Matching previous score
URVIN	0000100011001101	NA
FOLE	1111111110100010	NA
IH-1	1111111110011001	NA
OH-2	1101011100001011	Yes
KJON	0011111110111101	NA
VOLIN	1101000101011011	NA
JJN-3	0010000000111100	No – one digit difference*
INA-6	0011101011011000	Yes
ANBL-6	0101111010111101	Yes

C.

Name	Sex/age	M-protein	Ig	MM/PCL	Source
URVIN	M-52	IgGk	2.8 g/L	PCL, Terminal	Peripheral blood
FOLE	M-68	IgAλ	1 g/L	MM	Peripheral blood
IH-1	M-76	IgAλ	-	MM	Pleural effusion
KJON	F-66	IgGk	15 g/L	MM, Terminal	Peripheral blood
VOLIN	M-82	IgGλ	64.1 g/L	MM, Terminal	Bone marrow
OH-2	F-52	IgGk	80.3 g/L	MM, Terminal	Pleural effusion

Supplementary Figure 3

(A) Karyotyping of I: IH-1, II: URVIN and III: FOLE. The novel cell lines FOLE and URVIN, in addition to IH-1,were karyotyped by Giemsa-banding (G-banding). G-banding is a conventional cytogenetic method and was performed by standard procedures. All three cell lines' karyotypes are in line with non-hyperdiploid myeloma. For FOLE there were two distinguished karyotypes with different chromosomal numbers, but the main chromosomal aberrations are similar. Fluorescence *in situ* hybridization (FISH) was performed on metaphase spreads of IH-1, FOLE and URVIN and shows a t(4;14) translocation in all three HMCLs and also additional aberrations affecting t(4;14). 1p1q-FISH was performed in addition to characterize the deletion/amplification level in the cell lines. For FOLE, 1p16 (green) was missing corresponding to a deletion of the area. FISH was performed as

previously described[2]. (B) Binomial fingerprint score, using a fingerprint method[3], for each of the myeloma cell lines. NA: no previous score available. *The discrepancy could be explained by a different JJN-3 clone in our lab, as also briefly described in the manuscript. (C) Patient characteristics and clinical information at time of sampling for our in-house cell lines.

References

1. Skerget S, Penaherrera D, Chari A, et al. Comprehensive molecular profiling of multiple myeloma identifies refined copy number and expression subtypes. Nat Genet 2024;56(9):1878–1889.

2. Våtsveen TK, Tian E, Kresse SH, et al. OH-2, a hyperdiploid myeloma cell line without an IGH translocation, has a complex translocation juxtaposing MYC near MAFB and the IGK locus. Leuk Res 2009;33(12):1670–1677.

3. Keats JJ, Chesi M, Kuehl WM, Bergsagel PL. A Simple and Reliable Method To Verify the Authenticity and Purity of Human Myeloma Cell Lines. Blood 2007;110(11):2485.
